# Supplementary material for: A Fresh Look on Old Clothes: Laundry Smell Boosts Second-Hand Store Sales
Source: Brain Sci. 2022 Nov 10;12(11):1526. doi: 10.3390/brainsci12111526 (PMC9688905; doi:10.3390/brainsci12111526)
Supplement: Supplementary file 1 [file brainsci-12-01526-s001.zip › brainsci-2006911-supplementary.pdf]

Radboud Universiteit

English

Please rate your current feeling

Very negative — Very positive

1 2 3 4 5 6 7 8 9 10

Please rate your current energy level

Very low — Very high

1 2 3 4 5 6 7 8 9 10

Please rate your general impression of the store

Very negative — Very positive

1 2 3 4 5 6 7 8 9 10

Please rate the quality of the products

Very low — Very high

1 2 3 4 5 6 7 8 9 10

Please rate the cleanliness of the store in general

Not at all clean — Very clean

1 2 3 4 5 6 7 8 9 10

Please rate the cleanliness of the products

Not at all clean — Very clean

1 2 3 4 5 6 7 8 9 10

How much money did you spend in the store today?

- ○ I spent (in numbers, for instance: 25,10)

  [ ]

- ○ I prefer not to answer

What kind of products did you buy?

- ☐ Shirts
- ☐ Shoes
- ☐ Underwear
- ☐ Jackets
- ☐ Pants
- ☐ Accessories
- ☐ Blouses
- ☐ Other

How many products did you buy?

[ ]

How likely is it that you will visit this store again?

Very unlikely									Very likely
1	2	3	4	5	6	7	8	9	10

I identify as

- ○ Female
- ○ Male
- ○ Other
- ○ I prefer not to answer

My age is

- ○ Insert your age below (in numbers)

  [ ]

- ○ I prefer not to answer

Did you notice anything unusual in this shop? You can select multiple answers.

- ☐ I noticed a particular color or light
- ☐ I noticed a particular sound
- ☐ I noticed a particular smell
- ☐ I noticed a particular message
- ☐ I noticed nothing unusual in the shop

Radboud Universiteit

English

Please rate your overall smelling ability

Very bad — Very good

1 2 3 4 5 6 7 8 9 10

How would you describe the smell in the store?

- ○ Insert your description below
- ○ The store had no particular smell

Please rate the smell in the store on the following aspects:

Very low — Very high

1 2 3 4 5 6 7 8 9 10

Pleasantness (how nice the smell is)

Cleanliness (how clean the smell is)

Appropriateness (how the smell fits in the environment)

Intensity (how strong the smell is)

Familiarity (how known the smell is)
